# Residual Tensile Strength and Bond Properties of GFRP Bars after Exposure to Elevated Temperatures

**DOI:** 10.3390/ma11030346

**Published:** 2018-02-27

**Authors:** Devon S. Ellis, Habib Tabatabai, Azam Nabizadeh

**Affiliations:** 1 Ellis Engineering & Construction Management Services, LLC, Milwaukee, WI 53212, USA; dellis@ellis-engineering.com; 2 Department of Civil and Environmental Engineering, University of Wisconsin, Milwaukee, WI 53211, USA; azam@uwm.edu

**Keywords:** temperature effects, post-fire strength, glass fiber reinforced polymer bars, construction materials, reinforced concrete, high temperature properties

## Abstract

The use of fiber reinforced polymer (FRP) bars in reinforced concrete members enhances corrosion resistance when compared to traditional steel reinforcing bars. Although there is ample research available on the behavior of FRP bars and concrete members reinforced with FRP bars under elevated temperatures (due to fire), there is little published information available on their post-fire residual load capacity. This paper reports residual tensile strength, modulus of elasticity, and bond strength (to concrete) of glass fiber reinforced polymer (GFRP) bars after exposure to elevated temperatures of up to 400 °C and subsequent cooling to an ambient temperature. The results showed that the residual strength generally decreases with increasing temperature exposure. However, as much as 83% of the original tensile strength and 27% of the original bond strength was retained after the specimens were heated to 400 °C and then cooled to ambient temperature. The residual bond strength is a critical parameter in post-fire strength assessments of GFRP-reinforced concrete members.

## 1. Introduction

### 1.1. Background

Corrosion resistance and a superior strength-to-weight ratio are among the beneficial properties that have generated interest in fiber-reinforced polymer (FRP) reinforcing bars as a viable replacement for steel bars in reinforced concrete structural members [1]. Common FRP bars include glass fibers (GFRP), carbon fibers (CFRP), or aramid fibers (AFRP) in a polymer resin matrix. The fiber strands provide the primary tensile strength for the bars, while the polymer resin matrix serves as the binder. GFRP bars are the most commonly used FRP bars for civil-structural applications and are generally used in non-prestressed concrete members [2]. The application of GFRP bars in concrete is addressed in several standards, [2,3,4,5].

The mechanical properties of FRP reinforcement differ from steel reinforcement. FRP materials exhibit linearly elastic behavior and fail in a brittle manner. The compressive and shear strengths of FRP materials are dependent on the resin strength and are generally significantly lower than their corresponding tensile strength [3]. For this reason, the ACI 440.1R-15 document [3] recommends the use of FRP reinforcing bars only for conditions in which the bar is in tension. The bond strength of the FRP reinforcing bar is influenced by the resin material and by the reinforcing bar surface condition [6,7]. 

The mechanical properties of all common structural materials are adversely affected, to varying degrees, by exposure to high temperatures associated with fire. However, strength, stiffness, and bond properties of FRP reinforcements are affected more adversely by elevated temperatures when compared to steel reinforcing bars [1,3,8]. Many structures do not collapse after being exposed to fire. In such cases, a determination of the post-fire load-carrying capacity of the structure would be required. In reinforced concrete elements, the post-fire strength is dependent on the residual mechanical properties of the concrete and the reinforcement, including the residual bond between them. Similarly, to determine the post-fire strength of a GFRP-reinforced concrete member, the residual mechanical and bond properties of GFRP bars must be understood.

Available data indicate that the bond between the FRP bar and concrete is reduced at high temperatures [9], but little information exists on the extent to which the bond strength could be restored after cooling. It is also known that the tensile strength and the modulus of elasticity of the common FRP reinforcing bars decrease as the bar temperature is elevated [10], but the extent to which these properties recover after cooling requires additional investigation. Therefore, the primary objectives of this study were to assess the tensile and bond strengths and modulus of elasticity of GFRP reinforcing bars following exposure to elevated temperature and subsequent cooling to ambient temperature. 

### 1.2. Basic Mechanical Properties of GFRP

The properties of a FRP composite material are dependent on the fiber, the polymer matrix, and the relative proportions of the fiber, known as the fiber-volume ratio. The tensile strength and stiffness of the composite in the direction of the fiber are a function of the fiber material and the fiber-volume ratio. On the other hand, resin properties influence the compressive strength, shear strength, and bond characteristics of the composite [11]. Matrix properties also dominate the strength and stiffness in a direction transverse to the fibers. 

In addition to fiber and matrix properties, the size of a bar also affects its mechanical properties [12]. The rate of curing, the manufacturing process, and the manufacturing quality further influence the mechanical properties [11]. The ACI 440.1R-15 document [3] recommends that properties used for the design of GFRP reinforcing bars be those specified by the manufacturer. The guaranteed ultimate tensile strength and modulus of elasticity for the 19 mm bars used in this study were 620.5 MPa and 40,820 MPa, respectively [12]. The GFRP bars consisted of continuous E-glass fibers in a vinyl-ester resin matrix with a minimum fiber-volume fraction of 70 percent. GRFP bars used as reinforcement in structural concrete typically include deformations or other surface treatments to improve the bond with concrete. 

GFRP bars exhibit different longitudinal and transverse thermal expansion properties. The coefficient of thermal expansion of GFRP compares reasonably well with that of the concrete in the longitudinal direction. However, it could be as high as twice that of concrete in the transverse direction [3]. The coefficients of thermal expansion of GFRP range between 6.0 and 10.0 × 10^−6^/°C in the longitudinal direction, and between 21.0 and 23.0 × 10^−6^/°C in the transverse direction [3].

Drastic degradation of the post-fire mechanical properties of polymer composites occurs as the intensity or duration of the fire increases. The degradation of the mechanical properties of the FRP materials with non-organic fibers, such as glass fibers, relate to the decomposition of the polymer matrix [13]. When the resin in the FRP material is exposed to temperatures exceeding its glass transition temperature (T_g_), the polymer softens, resulting in a lower elastic modulus. The value of T_g_ is dependent on the type of resin but is typically between 65 °C and 120 °C [3]. Because of the reduction of force transfer capability between the fibers (due to the softened resin), the tensile strength of the overall FRP composite is reduced at temperatures above T_g_. Wang et al. [8] investigated the effect of elevated temperatures on the mechanical properties of 12.7-mm GFRP bars with a polyester matrix. The tensile tests showed reductions of 15%, 45%, 50%, 65%, and 90% (when compared to 20 °C results) at 100, 200, 300, 400, and 500 °C, respectively.

Robert and Benmokrane [14] investigated mechanical properties of FRP bars under temperatures of up to 315 °C. Results showed a reduction in tensile, shear, and flexural strengths, as well as modulus of elasticity. Da Costa Pires [13] investigated mechanical behavior of GFRP profiles under temperatures of 20 °C to 250 °C. The authors concluded that shear and compressive strength reductions at temperatures above the glass transition point were more significant than the tensile strength reduction. Kashwani and Al-Tamimi [15] also evaluated the behavior of GFRP bars under high temperatures, using two different groups of GFRP bar samples. The experiment showed a temperature of 350 °C as the critical temperature at which tensile strength decreased significantly.

### 1.3. Bond of GFRP with Concrete

Three main mechanisms influence the development of bond stress between FRP reinforcing bars and concrete [6,7,16,17,18]. These are: (1) the chemical bond or adhesion; (2) the friction between the FRP bar surface and the concrete; and (3) the mechanical interlock at the FRP surface with concrete. At the start of bar pull-out, adhesion is the main mechanism of stress transfer. However, since bond strength due to adhesion is relatively low due to the water repellent nature of FRP rebars [19], friction and mechanical interlock become the primary mechanism(s) for bond stress transfer at higher loads [17].

The difference in the transverse thermal expansion coefficient of concrete and FRP rebars can cause thermal stresses at contact surfaces of concrete and FRP rebars. As these thermal stresses increase up to the tensile strength of concrete, microcracks occur, resulting in bond loss and reduction in structural capacity. However, experimental results have shown that additional concrete cover may significantly mitigate the reduction in structural capacity due to fire exposure [20,21,22,23].

Okelo and Yuan [7] performed experiments to study the bond strength of GFRP, CFRP, AFRP, and steel reinforcing bars in normal-weight concrete. The authors reported that the bond strength was most influenced by concrete strength and the surface characteristics of the reinforcing bar. They concluded that the pull-out strength was not proportional to the embedment length, and the average bond strength decreased with the increasing bar diameter. Furthermore, the FRP bond strength was typically less than the bond strength for steel reinforcement, and the fiber type appeared to have an influence on the FRP bond strength. Malvar [6] reported that, given the same confining pressure, the bond strength for a steel reinforcing bar was, on average, 1.2 to 1.5 times larger than the bond strength of an equivalent GFRP reinforcing bar.

Masmoudi et al. [24] studied temperature effect on GFRP and steel bars experimentally and analytically. Samples were exposed to temperature increase up to 80 °C. No significant change in average bond strength was observed at the GFRP/concrete interface up to 60 °C. However, at a temperature of 80 °C, the average bond strength decreased 22% and 28% for 8 mm and 16 mm bars, respectively. 

Katz et al. [1] conducted pull-out bond tests on several commercially available GFRP reinforcing bars to investigate bond properties at temperatures ranging from 20 °C to 250 °C. The specimens were tested while at an elevated temperature. The behavior of the four types of 12.7 mm-diameter bars, with different surface treatments was compared with the steel reinforcing bar. At room temperature, all bars had bond strengths of 11 to 13 MPa, except for one FRP bar that had an unusually thick external polymer layer. Major reductions in FRP bond strength occurred at 180–200 °C. The tests also showed that the bond modulus (the slope of the ascending portion of the bond stress-slip curve) decreased as exposure temperature increased. The authors concluded that polymeric surface deformations would degrade at higher temperatures, leaving only the friction mechanism to resist bond stresses. Other studies have reported the temperature effect bond strength and mechanical properties of FRP rebars [13,14,25,26,27,28]. 

### 1.4. GFRP-Reinforced Concrete Members

The degradation of the mechanical properties of the FRP rebars with exposure to elevated temperature causes a reduction in the structural capacity of FRP-reinforced concrete members [29]. Bai et al. [22] conducted fire endurance experiments on full-scale GFRP slab specimens to study their response to sustained service level loads while exposed to an ISO-834 fire test (consisting of a temperature increase to 1100 °C in 180 min). Two specimens were subjected to four-point loading prior to, during, and after fire exposure. The authors reported that both specimens exhibited an average loss of flexural stiffness of 56% during fire exposure, but nearly one third of that lost stiffness was recovered after the specimens cooled to ambient temperature conditions. 

Abbasi and Hogg [30] conducted tests on two full-scale GFRP-reinforced concrete beams to investigate the behavior of the beams when exposed to fire. The beams were 350 mm × 400 mm × 4400 mm long. The tensile reinforcement for one of the beams (Beam 1) consisted of 12.7 mm GFRP reinforcing bars with vinyl ester resin, while the other beam (Beam 2) had the same GFRP bar size with a polyurethane matrix. The beams were subjected to four concentrated sustained loads of 10 kN each. Flexural failure of Beam 1 and 2 occurred after 128 min and 94 min of fire exposure, respectively. The corresponding average bottom reinforcing bar temperatures at the time of cessation of the heating for beams 1 and 2 were 462 °C and 377 °C, respectively. 

### 1.5. Research Significance

The study reported in this paper provides experimental data on the strength and stiffness of GFRP bars with prior exposure to high temperatures. The results can form the basis for estimating the residual post-fire strength of GFRP-reinforced concrete flexural members using analytical models. The effects of prior exposure to elevated temperatures on residual tensile strength, modulus of elasticity, and bond between concrete and GFRP bar are reported.

## 2. Materials and Methods

The GFRP bar used in the test specimen was a commercially available product, “Aslan 100” [12] (Hughes Brothers, Seward, NE, USA). The bar consists of continuous e-glass fibers (70% fiber volume ratio) in a vinyl ester resin. Two types of tests are discussed in this paper. The first type addressed the residual tensile strength of bars after exposure to high temperature. The second type focused on bond pull-out strength of GFRP bars in concrete after exposure to high temperatures. Table 1 shows the number and type of each test performed. The maximum test temperature was 400 °C, as measured at the surface of the GFRP bar. Previous research [8] indicated that the reinforcing bar would likely lose more than 60 percent of its tensile strength at this temperature.

### 2.1. Tensile Strength Tests

**Figure 1:**
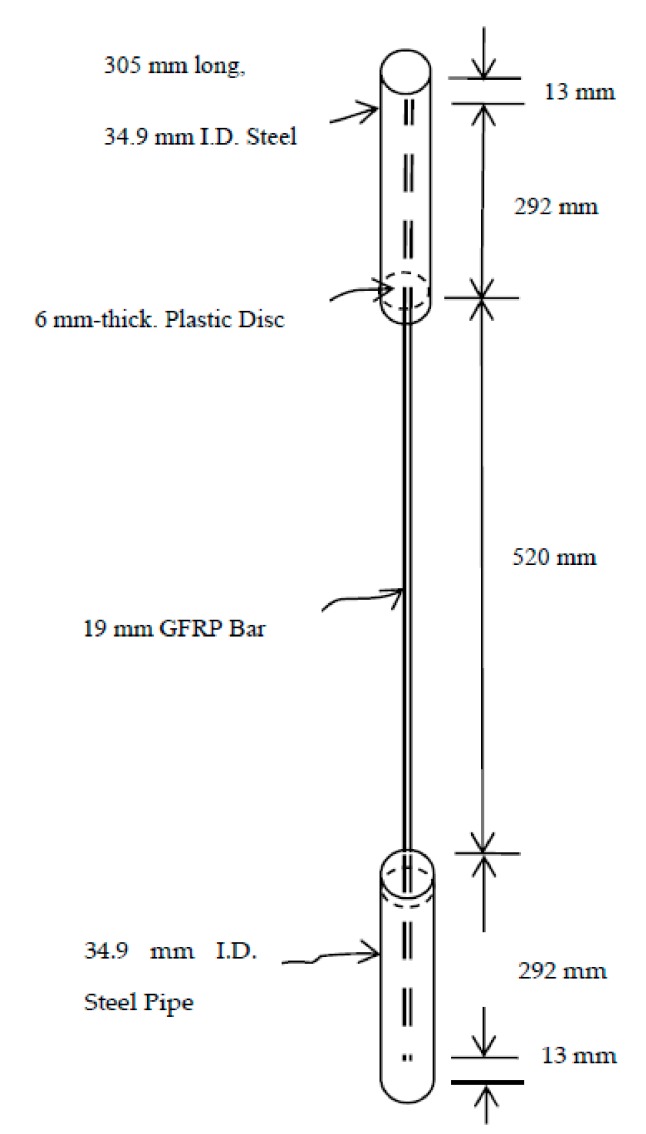
Tensile Test Specimen.

**Figure 2:**
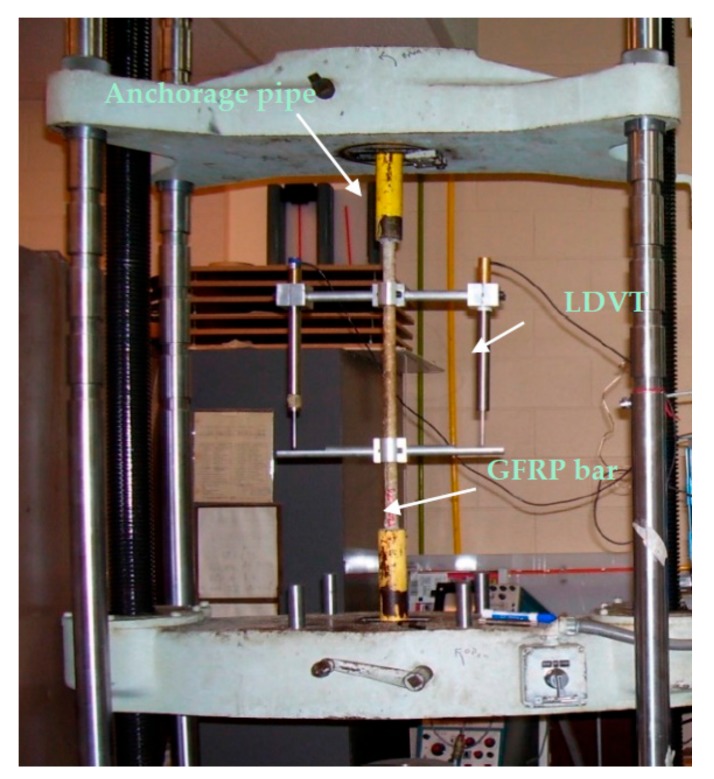
Tensile Test set up.

The tensile testing procedures were based on ASTM D7205 [31] and Castro and Carino [32] test procedures. Each specimen consisted of a 19-mm-diameter GFRP reinforcing bar with both ends embedded to a depth of 286 mm in a 305-mm-long, 38-mm-diameter (1½-inch) steel tube filled with a high-strength mixture of expansive cement, sand, and water. The choice of specimen length was to ensure that the rupture of the specimen occurred within the free length of the bar while considering the constraints of the testing equipment. Figure 1 and Figure 2 show details of the tension test specimens. 

To determine the tensile strength of the GFRP reinforcing bar at ambient temperature, one set of tensile test specimens (T-A) was placed in a universal testing machine at ambient temperature and loaded incrementally until the specimen ruptured. The universal tension-compression test machine was a screw-driven Tinius Olsen machine. To determine the stress-strain behavior of the bar material, two linear variable differential transformers (LVDT) were attached to the bar such as to measure the extension of the bar within the middle third of its free length, and the center-to-center distance between attaching arms (gage length) was recorded. The specimen was then loaded at a rate of about 8.9 KN/min until the bar ruptured. To prevent damage to the LVDTs, the devices were removed prior to the rupture of the rebar so the strain at rupture of the rebar in the specimens was not measured, and only partial displacement data were obtained for the tension specimens.

A heating tape was wrapped around the free length of the other TS specimens after placement of the bar in the test machine. Two thermocouples were attached to the bar surface under the heating tape. The free length of the bar was further wrapped with a ceramic insulation material. The specimen was loaded to 80.0 kN or 42% of the average rupture load obtained from the tests performed at ambient temperature. This load level was meant to represent the applied service load at the time of fire. The target service load was applied and held constant before heating the specimen to the desired temperature (within its free length). The heat was supplied to the specimens using custom-built equipment that consisted of a temperature controller and heating tapes capable of supplying a heat flux of 20.16 kW/m^2^ and a maximum temperature of 760 °C. The temperature at the surface of the reinforcing bar was measured at 5-min intervals during the heating. After the attainment of the desired temperature, the heat was turned off and the specimen was unloaded and removed from the tensile testing machine and allowed to cool down to ambient temperature in the laboratory for at least 24 h. Then, the specimens were placed back into the testing machine, the LVDT’s were attached, and the specimens were loaded until the bar ruptured.

### 2.2. Bond Pull-out Tests

**Figure 3:**
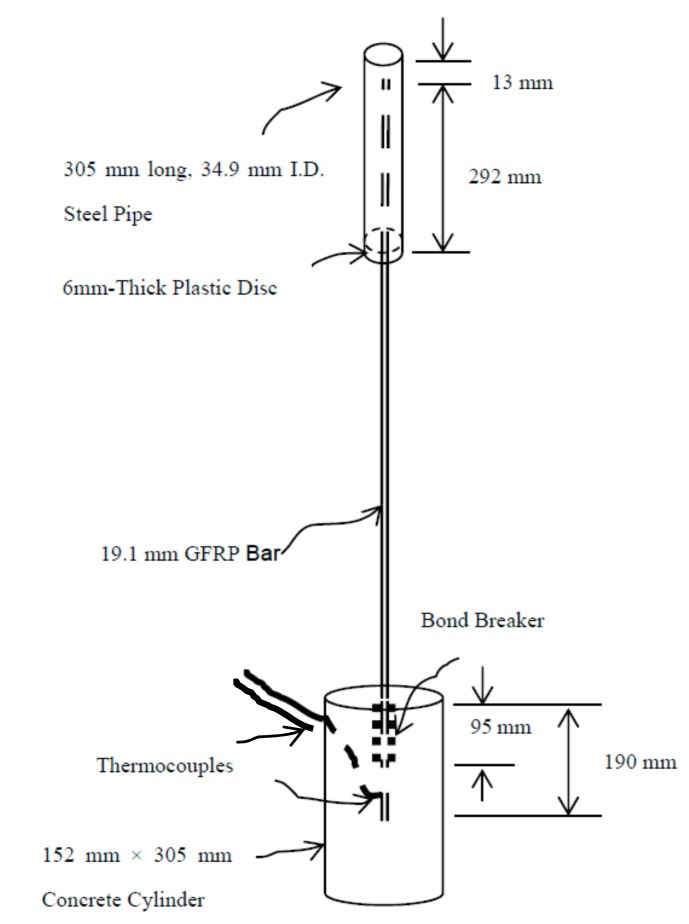
Bond Test Specimen.

Details of the bond test specimens were based on the ASTM D7913 [33] and similar setups used in earlier experiments by others [1,6,7,18], which is in accordance with test procedure. The specimen consisted of a 19-mm GFRP bar with one end embedded to a depth of 95-mm—or five times the bar diameter—at the center of a 152-mm diameter by 305-mm high concrete cylinder. Consistent with earlier experiments [1,7], the actual embedded length of 95-mm started beyond a 95-mm bond breaker created by wrapping the FRP bar with electrical tape (Figure 3). This was to minimize the effect of confining stresses due to the test setup. The opposite end of the bar was identical to the ends used on the tensile test specimen (Figure 3).

The concrete used in the cylinder was designed to achieve a 28-day compressive strength of 27.6 MPa. The specimens had a high-temperature thermocouple embedded in the concrete such that the thermocouple touched the surface of the reinforcing bar at a depth of 143 mm from the top surface of the concrete (middle of the bonded height of the reinforcing bar). 

**Figure 4:**
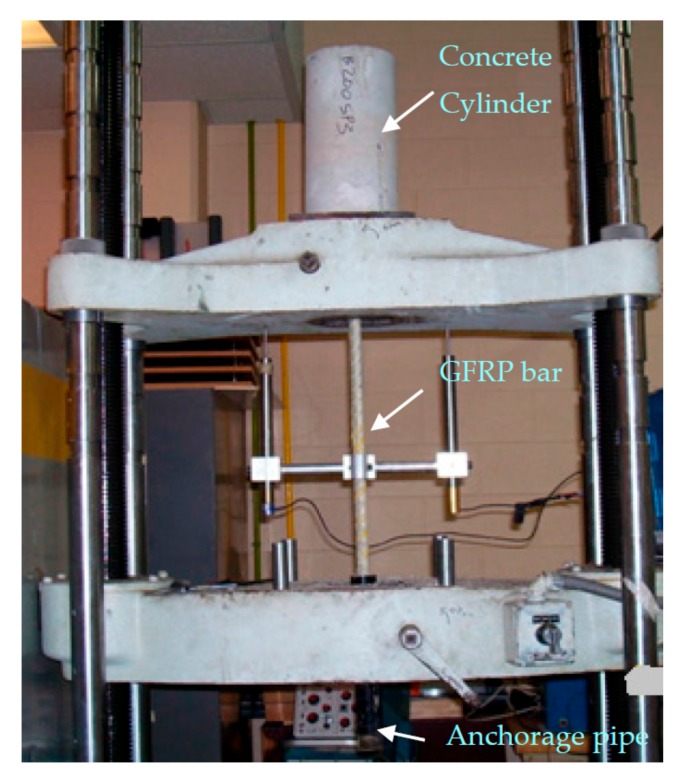
Bond Test set up.

To perform the bond tests, the specimens were placed in the universal testing machine with the concrete cylinder bearing on the top cross-head of the machine and the gripping end of the specimen in the grips in the lower cross-head (Figure 4). LVDTs were attached to the reinforcing bar with purpose-built brackets. The “B-A” specimens were loaded incrementally at ambient temperature until bar pull-out or any other form of specimen failure occurred. Failure was deemed to have occurred when the load reading from the tensile testing machine started to drop. During the loading process, the corresponding slip was measured by LVDTs. The bond force-slip relationship and the force at pull-out were recorded, and the bond strength at ambient temperature was determined. 

For B-100, B-200, and B-400, the concrete cylinder was heated while the specimens were in an unloaded state and lying horizontally on ceramic insulation on the laboratory floor. The cylinders were not heated under full “service loading”, because the first specimen heated under such loading failed (pulled out) when the temperature reached 85 °C. The failure of the bond specimen under the maximum service load (at 85 °C) indicates that the bar development length used in design calculations should be adjusted for the effect of elevated temperatures because of its significant influence. However, in common reinforced concrete beams, the length from the point of maximum moment (typically mid-span) to the end of bar (bond length) may be larger than the required bar development length, with or without elevated temperatures. The failure that was observed in the bond test specimen occurred under the full service-load over a short embedment length (resulting in high bond stresses). The focus of this study was on residual post-fire behavior, assuming survival during the fire. Therefore, if the bond failure did not occur and the structure survived, the bond stresses were necessarily lower than the corresponding bond strength because of low axial loads, long bond length, and/or low temperature exposure. Therefore, to allow for the assessment of the residual bond given survival after a fire, the bond test specimens were heated without a service axial load applied. 

The same special-built heating equipment that was used in the tensile tests provided the heat for the bond tests. The specimens were heated by wrapping the heating tape around the concrete cylinder. All perimeter concrete surfaces, and the heating tape wrapped around it, were covered with ceramic insulation prior to the commencement of heating. A thermocouple was placed at the surface of the concrete at the location of the heating tape so that the corresponding concrete surface temperature could be monitored while the temperature at the reinforcing bar was also being recorded. Another thermocouple was placed inside the concrete at the surface of the bar. To accommodate the temperature increases due to thermal lag, the temperature controller was set to shut the heat supply off when the reinforcing bar temperature was 15 °C less than the desired temperature. After the desired temperature at the location of the reinforcing bar was attained, the insulation and heating tape were removed from the specimens, and the specimens were left in the laboratory to cool to ambient temperature. 

After cooling naturally in the laboratory for at least 24 h, the specimens were placed in the tensile testing equipment as shown in Figure 4. Loading was then applied until bar pull-out occurred. The force-slip relationship and the force at pull-out were recorded. 

## 3. Results

### 3.1. Tensile Test Results

**Figure 5:**
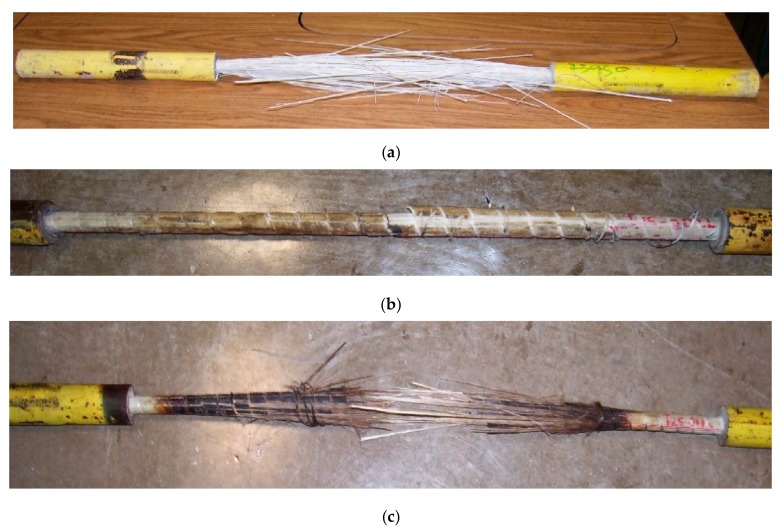
Tensile test specimens after failure: (**a**) T-A, (**b**) T-200, (**c**) T-400.

Figure 5a shows a photograph of a T-A specimen after failure. The failure of the T-200 specimens was characterized by large longitudinal cracks in the rebar and the breaking away of the outer layer of the bar (Figure 5b). This contrasts with the failure characteristics observed in the T-A and T-100 specimens, in which the fibers appeared to explode away from the bar. Failure of the T-400 specimens was less explosive than that observed in the lower temperature tests, and all T-400 specimens ruptured at failure (Figure 5c). Table 2 summarizes the results from the tensile strength tests.

### 3.2. Bond Test Results

**Figure 6:**
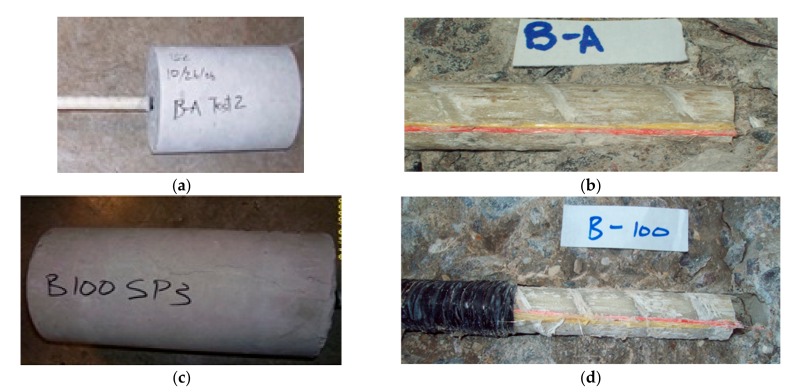

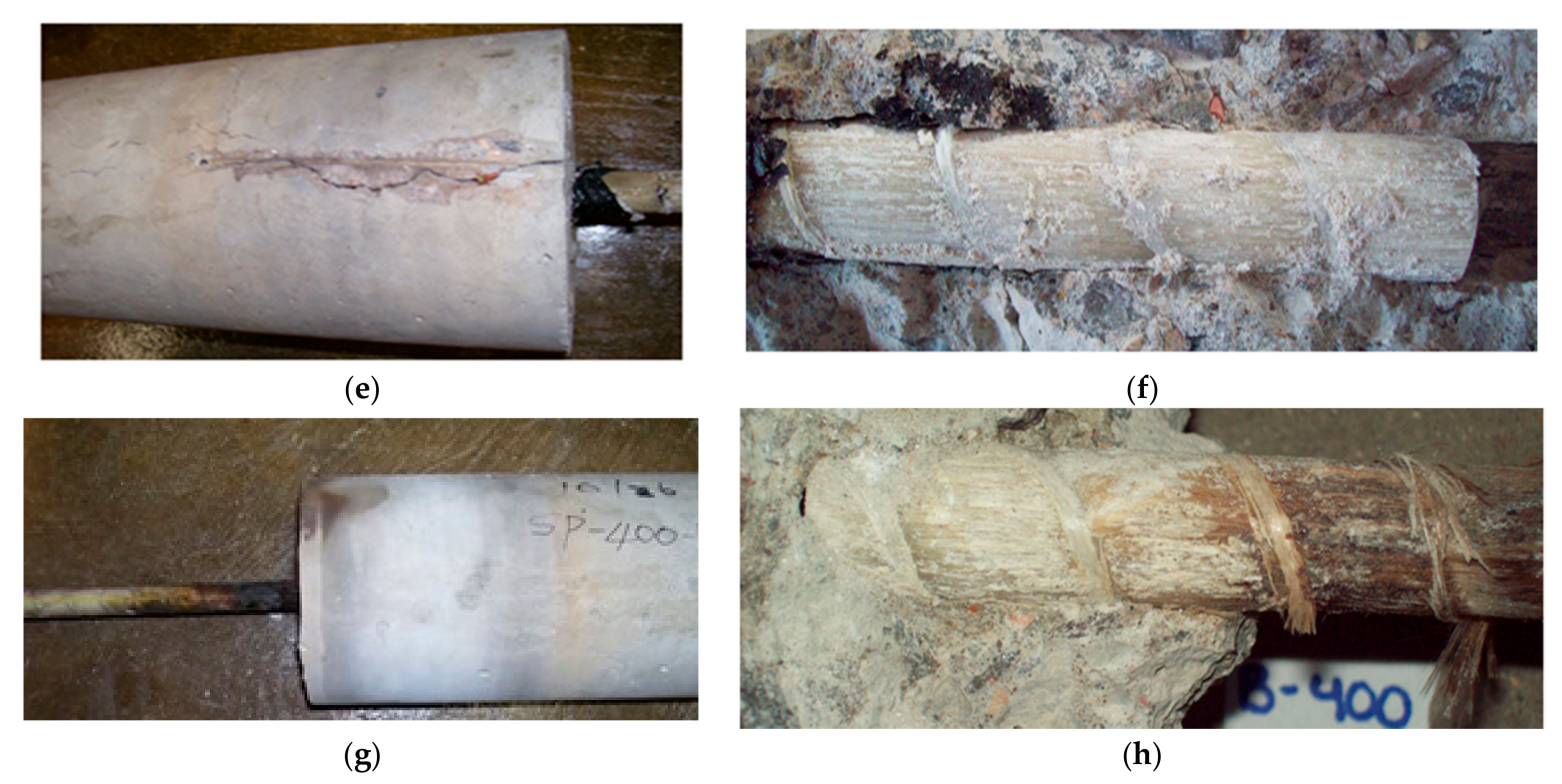
The bond specimens after bond failure. (**a**) Side view of B-A specimen after bond failure; (**b**) B-A specimen showing embedded portion of bar after failure of specimen; (**c**) side view of B-100 specimen after bond failure; (**d**) B-100 specimen showing embedded portion of bar after failure of specimen; (**e**) side view of B-200 specimen after bond failure; (**f**) B-200 specimen showing embedded portion of bar after failure of specimen; (**g**) side view of B-400 specimen after bond failure; (**h**) B-400 specimen showing embedded portion of bar after failure of specimen.

Each of the B-A specimens (bond specimens that were not exposed to elevated temperature) failed by pull-out of the rebar from the concrete cylinder and no cracking of the concrete was observed (Figure 6a). Some resin and glass fibers were observed on the concrete (Figure 6b).

After the heating, tapes were removed from the B-100 specimens; small random cracks, forming a web-like pattern, were observed on the concrete surface that had direct contact with the heating tape. Failure of all three of the B-100 specimens was by pull-out of the rebar from the concrete. However, unlike the unheated specimens, failure of the B-100 specimens was associated with cracks that radiated from the rebar to the surface of the concrete cylinder (Figure 6c). Additionally, when the concrete was broken after pull-out failure had occurred, significant damage to the bar surface was apparent and resin (Figure 6d) and glass residue was observed on the concrete.

In all cases, failure of the B-200 specimens occurred by pull-out of the rebar from the concrete cylinder. Failure involved cracks (smaller than those in the B-100 specimens) radiating from the rebar location to the surface of the concrete cylinder (Figure 6e). Significant damage to the bar surface was apparent, and resin and glass residue was observed on the concrete surface that was in contact with the bar (Figure 6f). However, there was more resin residue and less glass residue observed on the concrete in the B-200 specimens compared to B-100 specimens. 

One of the B-400 specimens was damaged during test setup. Consequently, only two B-400 specimens were available for complete testing. There was significant discrepancy between the failure loads observed for the two specimens. However, both specimens failed by rebar pull-out and there was no evidence of any cracking in concrete (Figure 6g). Significant damage was observed on the bar surface, including charring of the upper part of the embedded portion of the bar (Figure 6h). Some resin residue was observed on the lower portion of bar, but the bar surface generally appeared to be depleted of resin. 

**Figure 7:**
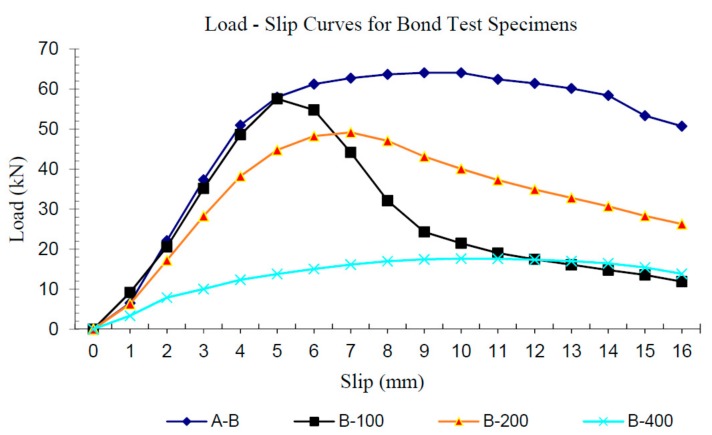
Comparison of mean load-slip curves for bond test specimens.

Table 3 provides a summary of the results from the bond tests and Figure 7 provides a graphical comparison of the load-slip relationships obtained from the specimens tested. A substantial reduction in the residual bond strength was observed for the specimens that were exposed to 400°C.

## 4. Discussion

### 4.1. Discussion of Tensile Test Results

The average ultimate tensile strength of 649 MPa, obtained from the T-A tests, compares favorably with the guaranteed ultimate strength of 621 MPa provided in physical properties data published by the manufacturer [12]. Also, the mean modulus of elasticity of 39,580 MPa obtained from these tests is only 2.9% lower than the value provided by the manufacturer. In summary, the test results for the T-A specimens were consistent with the published data and validate the specimen design and test procedure for the T-A specimens.

The 87% residual strength for T-100 specimens appears to be consistent with the findings of Wang et al. [8] for tests under elevated temperatures. The peak temperature to which the T-100 specimens were exposed (100 °C) is very close to the glass transition temperature of the bar’s vinyl ester resin matrix [34]. It is therefore reasonable to expect that the resin would exhibit some reduction in properties. 

Also of interest is the fact that the average strength of the T-200 specimens was 11% higher than that of the T-100 specimens and only 2% lower than the average strength of the T-A specimens. 

The high strength of the T-200 specimens relative to the T-100 specimens may be explained in terms of a phenomenon called post-cure (further molecular cross-linking) that has been observed at temperatures between 100 °C and 150 °C by Mouritz and Gibson [34]. Post-cure increases the polymer’s molecular weight and improves its mechanical properties.

As with the other tensile test specimens, all T-400 specimens ruptured within the free length of the bar, thereby providing validation for the test setup. The fact that one of the specimens ruptured during heating while subjected only to the sustained load raises questions about reliability of the bar when it is exposed to temperatures above 400 °C. 

### 4.2. Discussion of Bond Test Results

The mean bond strength of 11.25 MPa obtained from the tests of the B-A specimens compares favorably with the bond stress of 11.57 MPa provided in the manufacturer’s literature [12]. Additionally, the mean total slip of 9.4 mm (corresponding to the maximum bond force) obtained for the B-A specimens compares reasonably well with the value of 7.9 mm indicated in the manufacturer’s product literature [12]. The load-slip curves for the B-A specimens are consistent with those in published literature [6,7,12,17]. The bond failure mechanism in the B-A specimens involved longitudinal slip within the bar near its surface. Resin and glass fiber residue were observed on the concrete. This is consistent with a failure mechanism that involves abrasion of the bar’s surface [7].

In B-100 specimens, the resin and glass residue observed on the concrete and the damage seen on the bar surface indicate that the failure mechanism also involved shear failure within the outer layer of the bar. The fact that more resin residue was observed on the concrete surface of the B-100 specimens is likely due to the reduced shear strength of the resin. This would also account for the 4% reduction in bond strength of the B-100 specimens relative to the B-A specimens. The concrete splitting (cracking) suggests the effect of the mechanical interlock mechanism [17]. 

The bond-slip curve for B-200 specimens showed lower strength and stiffness compared to the B-100 curve. The flatter slope on the linear portion of the load-slip curve and the lower bond strength observed for the B-200 specimens suggests lower shear stiffness and shear strength in the outer layer of the bar in the B-200 specimens. Also, more resin residue was observed on the concrete, providing more evidence of reduced resin shear strength. There were radial cracks in the concrete cylinder associated with the failure of the B-200 specimens. However, because of the reduced shear strength of the resin, smaller stresses are transferred to the concrete, thereby producing cracks with smaller widths and lower frictional forces compared to B-A and B-100 specimens.

During the tensile testing, improvement of the strength in the bar that was heated to 200 °C relative to the strength in the bar that was heated to 100°C was observed. This suggests an improvement in the resin properties. This contrasts with the reduction in shear strength of the resin implied by the reduced bond strength (relative to the B-100 specimens) observed in the B-200 specimens. That is, the post-cure effect was not observed in the bond specimens. It is possible that this is due to the moisture in the concrete, which is known to degrade the properties of resins, or it may be due to redistribution of bond stresses [3,35].

The load-slip response for the B-400 specimens (Figure 7) indicates that, because of the significant degradation of the resin at the surface of the bar, the “bond stiffness” and bond strength are significantly reduced. Therefore, stresses transferred to concrete are reduced, and cracking was not observed. These factors indicate that bond of FRP to concrete after the bar has been exposed to temperatures exceeding 400 °C cannot be reliable and predictable. Therefore, there could be important structural implications (i.e., structure may not be suitable for return to service) in such cases depending on estimated temperature exposure, as well as the sensitivity to bond strength in the structural configuration. 

**Figure 8:**
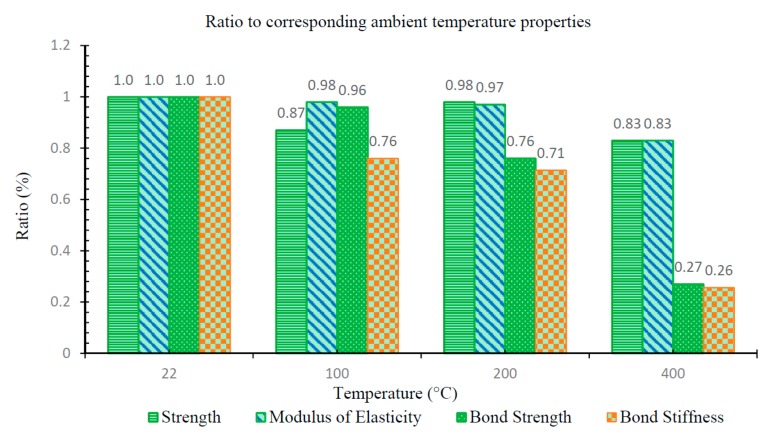
Ratios of residual parameters of pre-heated specimens with respect to their corresponding unheated test specimens.

Figure 8 shows relative reductions in residual tensile strength, modulus of elasticity, and bond strength of specimens that were previously exposed to elevated temperatures compared to tests performed on unheated specimens. This figure illustrates that the bond strength is the most critical parameter in assessing strength of GFRP-reinforced concrete beams that have survived fires, especially when GFRP bars were heated to temperatures of 200 °C to 400 °C before cooling. Although the bar’s residual tensile strength and modulus of elasticity also decreases with exposure to increasing temperatures, it is the residual bond stiffness and residual bond strength that exhibit drastic reductions.

## 5. Conclusions

Although substantial information exists regarding the performance of FRP bars under high-temperature conditions, data on residual tensile strength and bond (to concrete) properties after cooling are not widely available. In cases in which an FRP-reinforced concrete structure survives a fire, it is important to assess the residual strength properties. Experiments performed as a part of this study provide information on the residual tensile properties of GFRP bars after they have been exposed to elevated temperatures of up to 400 °C.

Further details of this research including analytical aspects are provided by Ellis [36].

The conclusions of this study can be summarized as follows:

- The residual tensile strength and modulus of vinyl ester GFRP bars generally decreases as the exposure temperature increases. However, the residual strength of the bar that was exposed to 200 °C was larger than the bar exposed 100 °C, which is attributed to the post-cure phenomenon.
- Vinyl ester GFRP bars retain a significant percentage of their tensile strength and elastic modulus after they have cooled from elevated temperatures of up to 400 °C. For example, after exposure to a temperature of 400 °C, the average residual strength of the bar (upon cooling) was 83% of its ambient temperature strength. This contrasts with the percentage of retained properties during exposure to elevated temperature. According to Wang et al. [8], GFRP bars retain about 35% of their ambient temperature strength while subjected to a temperature of 400 °C.
- Generally, the residual pull-out bond strength of the GFRP bars decreased as the exposure temperature increased. There was no significant change in bond stiffness with exposure temperatures of less than 100 °C. The mechanisms of bond transfer also change for different levels of temperature exposure. There is a drastic reduction in bond strength and bond stiffness as prior exposure temperatures reach 400 °C. This is believed to be a major factor affecting post-fire strength assessments of GFRP-reinforced concrete beams, and must be carefully considered in such assessments.

## Figures and Tables

**Table 1:** Test Parameters.

Specimen	No. of Tests	Test Type ^1^	Description
T-A	3	TS	Test at ambient temperature. No heat exposure.
T-100	3	TS	Test at ambient temperature after exposure to temperature of 100 °C at constant load.
T-200	3	TS	Test at ambient temperature after exposure to temperature of 200 °C at constant load.
T-400	3	TS	Test at ambient temperature after exposure to temperature of 400 °C at constant load.
B-A	3	PO	Test at ambient temperature. No heat exposure.
B-100	3	PO	Test at ambient temperature after exposure to temperature of 100 °C.
B-200	3	PO	Test at ambient temperature after exposure to temperature of 200 °C.
B-400	3	PO	Test at ambient temperature after exposure to temperature of 400 °C.

^1^ TS: Tensile strength of GFRP bar; PO: Pull-out bond test

**Table 2:** Summary of Tensile Test Results.

Specimen	Temperature Exposure, °C	Mean Failure Load, kN	COV (%) of Strength	Mean Residual Tensile Strength, MPa	Mean Residual Modulus of Elasticity, MPa	COV (%) of Modulus of Elasticity	Ratio of Residual Strength to Ambient Temperature Strength	Ratio of Residual Modulus to Ambient Temperature Modulus
T-A	22	191.7	4.1	648.9	39603	3.9	1	1
T-100	100	167.3	1.3	566.1	38969	2.2	0.87	0.98
T-200	200	187.3	4.2	633.8	38388	5.2	0.98	0.97
T-400	400	158.3	1.4	535.7	32839	3.1	0.83	0.83

**Table 3:** Summary of Bond Test Results.

Specimen Name	Temperature at Embedded Portion of Bar, °C	Mean Failure Load, KN	COV (%) of Failure Load	Mean Residual Bond Strength, MPa	Ratio of Residual Bond Strength to Ambient Temperature Bond Strength
B-A	22	64.2	7.9	11.3	1.00
B-100	100	61.9	13.3	10.9	0.96
B-200	200	48.6	4.6	8.5	0.76
B-400	400	17.6	64.7	3.1	0.27
